# Green Natural Colorants

**DOI:** 10.3390/molecules24010154

**Published:** 2019-01-02

**Authors:** Isabel Viera, Antonio Pérez-Gálvez, María Roca

**Affiliations:** Food Phytochemistry Department, Instituto de la Grasa (CSIC), University Campus, Building 46, Carretera de Utrera km. 1, 41013 Sevilla, Spain; iviera@ig.csic.es (I.V.); aperez@ig.csic.es (A.P.-G.)

**Keywords:** ADME, absorption, chlorophylls, chlorophyllin, green colorant, zinc-chlorophylls, copper-chlorophyll, coloring foodstuff, natural colorants, food colors

## Abstract

Although there is no legal and clear definition of the term “natural food colorant”, the market trends, and consequently industrial and commercial interest, have turned to foods with added natural pigments. This progressive substitution of artificial colorants has faced chemical complications with some colors, with a lack of stable green hues being one of them. Several strategies have been applied for green color stabilization in processed foods, from the formation of metallochlorophylls to the microencapsulation of green pigments. However, at present, the utilization of green coloring foodstuffs, which are considered an ingredient in the EU, seems to be the more successful solution for the market. Besides those topics, the present review aims to clarify the current confusion between the different chlorophyll compounds that form part of the authorized green food colorants. In this sense, legislations from different countries are compared. Finally, and in line with current concerns, the knowledge gathered so far in relation to the absorption, distribution, metabolism and excretion of all green natural food colorants is reviewed.

## 1. Introduction

The first moment of truth is a term coined by Procter & Gamble [1] to describe the 3–7-s gap that makes a customer select a product over the rest of its competitors. It has been established that color is responsible for 62–90% of the consumer’s assessment [2]. This fact makes expertise in food colorants a very profitable activity. Specifically, some market research companies have prognosticated a global food colors market size of USD 2.97 billion by 2025, with an estimated USD 1.79 billion in 2016 [3]. In this sense, consumer claims are responsible for the progressive strengthening of the food industry. We want food products to be more delightful, nutritive, attractive and healthy, with “fun-foods” as the maximum expression of this appeal [4]. The response to this worldwide trend is the unavoidable application of food colorants.

Before going further in this review, several concepts should be defined. Colorants can be classified following different parameters [5]. For example, in the USA, food colorants are categorized into whether they require or not the batch certification carried out by the US Food and Drug Administration (FDA). The list with the approved colorants that require certification is published in Title 21 CFR (Code of Federal Regulations) 74, where a Foods, Drugs and Cosmetics (FD & C) number is assigned. Hence, the certified colors commonly defined as synthetic could be classified as dyes (food-grade water soluble colorant [6]) or lakes (oil-dispersible; generally, dye extended on alumina). On the other hand, the list of the colorants exempted from certification is published in the Title 21 CFR 73 document [7]. Colorants within this category are mainly natural, although a few of them are produced by synthesis, but considered “nature-identical”. On the other hand, authorized food colorants in the EU are mainly legislated by the Regulation (EU) No. 1333/2008 [8], which is amended by Regulation (EC) No. 1129/2011. EU legislation includes indistinctly natural or artificial colorants.

Food colorants can be allocated following their origin (vegetal, animal, bacterial, fungal, etc.), their hue (red, yellow, purple, blue, green, etc.) or their chemical structure: flavonoid derivatives (anthocyanins), isoprenoid derivatives (carotenoids), nitrogen–heterocyclic derivatives (betalains), and the subject of this review, pyrrole derivatives or chlorophylls, which are responsible for blue and green hues.

At this point, it is relevant to distinguish between natural colorants and artificial ones. Although we could have an intuitive and clear conception about what each term means, the truth is that there is no official or definitive definition of what a natural or an artificial colorant is [9]. Only natural flavorings, another class of food additives, have a specific definition given by the FDA and EU [10]. Although there have been suggestions to extend the distinction of natural flavorings to food colorants, it seems difficult to reach a consensus between the different interested parties. In fact, neither in the US nor in the EU is there a recognized legal advertisement for “natural color” [11]. Indeed, the limits could be even more ambiguous if we consider, for example, the food colorant copper chlorophyllin, which is considered as natural. As will be noted below, this colorant is extracted from a natural source (generally from edible green leaves), but its manufacture requires additional chemical batch processing. Consequently, the question is as follows: where do we set the limit between natural and synthetic colorants? However, even realizing the hurdle of setting a frontier between natural and artificial colorants, the consumer increasingly demands more information, transparency, naturalness, clarity and trustworthiness in food label specifications. Thus, it is evident that there is an increasing global trend towards the natural side of foods.

Although previous studies linked the consumption of artificial food colorants with behavioral disorders, the inflection point was probably the well-known “Southampton study” [12]. This randomized, double-blinded trial test was performed with 137 3-year-old and 130 8/9-year-old children and concluded that the intake of dietary artificial food colorants increased the hyperactivity of children. Since then, many other research works and meta-analysis studies have confirmed and even amplified the disorders which probably originated from artificial food colors, although the precise physiological mechanism is unknown to date. In any case, the food industry and food legislative authorities have implemented some actions. The consumers’ concern about the safety of artificial food colorants, reinforced by the possible health benefits of natural pigments, have induced the food industry to withdraw artificial colorants. In fact, artificial food colorants represent only 16% in the EU and 29% in the North America of the food colorants portfolio [13]. 

The progressive substitution of synthetic colorants by natural ones faces the challenge of the low stability of some colorants. Probably, green is one of the most complicated colors to both naturally set-up and counteract. The aim of the present review is to specifically address the current status of natural green food colorants, including the legal issues, the chemistry of chlorophylls and the existing alternatives for the stabilization of green color in foods, as well as the acclaimed “coloring foodstuff”. For a general study of other food colorants, high-quality reviews have recently been published [4,10,11,13,14,15,16,17].

## 2. Legal Aspects on Green Natural Colorants

As has been noted elsewhere [16], one of the main problems affecting food additives is the lack of a globally harmonized legislation. This fact creates problems for wholesalers in this globalized food market. Even the concepts “natural” and “artificial additives” are country-dependent. Consequently, although this review only deals with the natural green colorants, whose main legal considerations are firstly described, a brief outline also shows the authorized synthetic green food colorants in different countries at the end of this section. Generally, the regulations of food additives include the list of authorized standards, their specifications, as well as the conditions of use (limitations on specific foods and maximum amounts). In addition, other requirements that should be considered are the schemes through which authorization is obtained and the conditions for packaging and labelling, which are different in each country.

**Figure 1:**
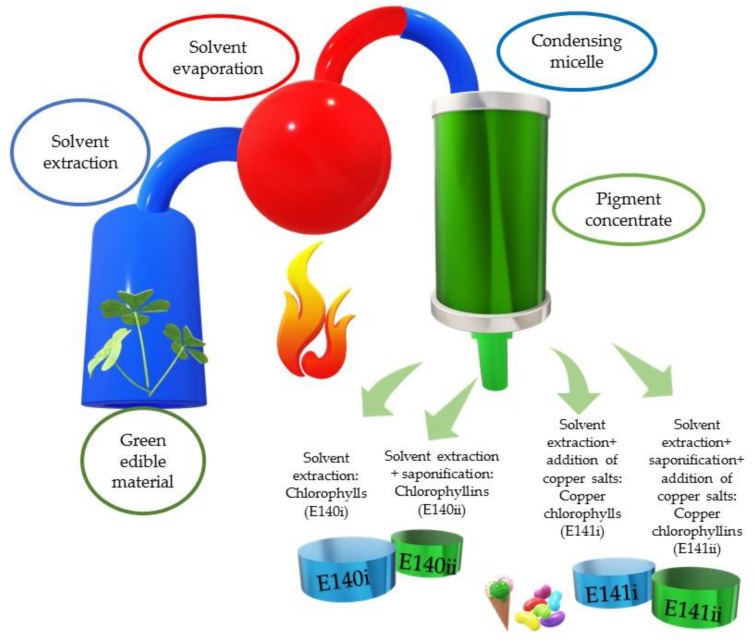
Scheme of the natural green colorant manufacture process.

European current legislation (Regulation (EC) No 1333/2008 and its amendments) [8] has allowed the use of two natural green colorants (Table 1), E140 and E141, which are structurally related with the chlorophylls, since the first European food colorant legislation was published in 1962 (Council Directive 62/2645/EEC). E140 comprises direct chlorophyll derivative extraction with organic solvent from natural sources: grass, alfalfa, nettles, spinach and other edible plant materials (Figure 1). The end-product could contain other lipids, pigments and waxes, so that the final aspect of the colorant is waxy, and it is marketed according to its solubility. Thus, the E140i or chlorophyll derivative is lipid-soluble, while the alternative for water-soluble foods (marketed as a powder) is E140ii (or chlorophyllins), obtained by the saponification of the solvent-extracted yield from edible plant material. Saponification breaks the ester–phytol bond, as will be detailed below, increasing the polarity of the derived products. However, both colorants are rather unstable, and the color is prone to experience a drastic change from green to brown. Therefore, the food industry favors the use of the colorant E141, which results from the addition of copper to the corresponding lipid or water-soluble chlorophyll solutions. The insertion of copper into the chlorophyll molecule stabilizes the structure, and the green coloration does not change, independent from the processing conditions or the storage time of the colored food. E141 is mainly composed of copper complexes of chlorophyll derivatives, with E141i (or copper chlorophylls) being the lipid-soluble option and E141ii (copper chlorophyllins) the water-soluble alternative [18].

In the USA, the regulation of natural food colorants is published in Title 21 CFR 73 [7]. Only copper chlorophyllin (Table 1) is authorized as a natural green colorant (CFR Section 73.125) and only for coloring citrus-based dry beverage mixes, the amount being limited to 0.2% in the dry mix. Current legislation in China [19] recognizes two green colorants: copper chlorophylls (CNS 08.153) and copper–sodium chlorophyllin (CNS 0.009), which is only allowed for use in determined food categories and with fixed maximum levels. At any rate, the China National Center for Food Safety Risk Assessment (CFSA) has published at the beginning of 2018 the first draft for a National Food Safety Standard for the Use of Food Additives, which revises and updates the current version issued in 2014. In Japan, the list of food additives from natural origin was compiled and published by the Ministry of Health and Welfare on 16 April 1996 [20]; in 2018, this accounts for 365 items, with chlorophyll (number 117 in the original Japanese list) and chlorophyllin (116) as the only authorized natural green colorants. In addition, Japanese legislation includes 455 designated food additives to date (obtained by chemical reactions), which include copper chlorophyll (266), sodium–copper chlorophyllin (265) and sodium–iron chlorophyllin (257). In both regulations, the target foods, the maximum limits and other requirements are detailed for each colorant. In India, the regulations are published in the Food Safety and Standards (Food Products Standards and Food Additives) Regulations (2011), which depend on the Food Safety and Standards Authority of India [21]. They only allow the use of chlorophyll as a coloring food matter which has the specification of having both chlorophyll *a* and chlorophyll *b*.

Finally, the Joint Food and Agriculture Organization of the United Nations/World Health Organization (FAO/WHO) Expert Committee on Food Additives (JECFA), as the international body responsible for evaluating the safety of food additives, has published the last version of the Codex Alimentarius general standard of food additives (41st meeting on 2018) [22]. In accordance with European legislation, chlorophylls (INS 140) and copper chlorophylls (INS 141) are registered. It is worthwhile to visit the corresponding websites to have access not only to the specification of each food additive, but also to the complete monographs and evaluations developed for each one.

**Figure 2:**
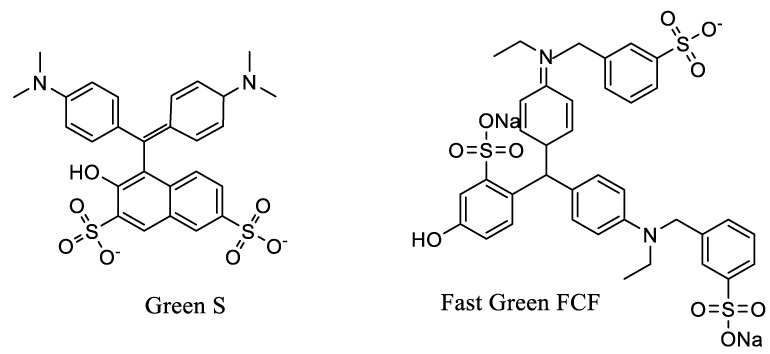
Structures of green S and fast green FCF.

Along with the colorants generally considered as natural, the legislations also authorize artificial green food colorants (Table 2), green S and fast green FCF, with both compounds being triarylmethane derivatives (Figure 2). In the EU, only green S is legal, under the code E142, while its use is banned in countries such as the USA, Japan, India, and China. It is also recognized by the Codex Alimentarius with the reference number INS 142. This colorant presents the molecular formula C_27_H_25_N_2_NaO_7_S_2_ and a molecular weight equal to 576.63 Da (CAS number 3087-16-9). On the other hand, in the USA (along with other countries, Table 2), the authorized artificial green colorant is the so-called fast green FCF, a compound with the molecular formula C_37_H_34_N_2_O_10_S_3_Na_2_ and a molecular weight equal to 808.85 Da (CAS number 2353-45-9; PubChem CID 73557432), while its use is banned in the EU.

## 3. Chlorophyll Derivatives in the Authorized Natural Green Colorants

**Figure 3:**
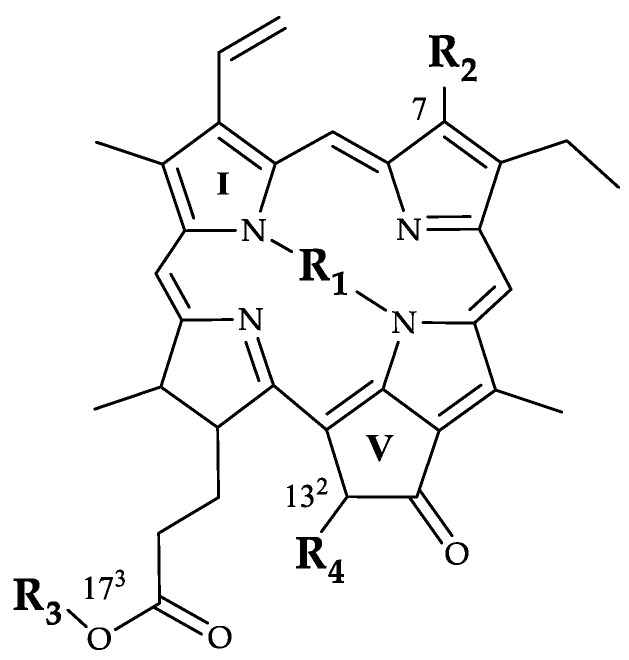
Chlorophyll skeleton with the different substituents.

Several chemical (conventional) terms have been commonly confused in the commercial and the industrial environments to tag chlorophyll colorants. We will explicitly detail the chlorophyll compounds, when described, that make up each natural green colorant, along with the conventional terms. To avoid misunderstanding in following the statements, Figure 3 depicts the skeleton of the chlorophyll structure showing the different substituent functions and the numbering system. The difference between chlorophyll *a* and chlorophyll *b* is located at C7, where chlorophyll *a* presents a methyl group while chlorophyll *b* includes a formyl group (Table 3). The central coordinated atom is magnesium in the chlorophylls, while two hydrogen atoms are present in pheophytins and pheophorbides. Otherwise, the phytyl chain (C_20_H_40_) is ester-bonded to the C17^3^, which confers the lipophilic properties when present in non-polar derivatives: chlorophylls and pheophytins (Table 3). On the opposite set, the phytyl chain is absent in polar derivatives: chlorophyllides and pheophorbides. Lastly, the isocyclic ring (ring V) presents a carboxymethoxy arrangement at C13^2^. The lack of this group yields the pyroderivatives. Additional unprecedented chlorophyll structures could be present in the colorants [23].

The analysis of the chlorophyll derivatives contained in the green natural colorants started with the development of several chromatographic methods able to separate the different compounds in the mixtures [24,25]. The details of those methods have been recently compiled [23]. Due to the chromatic properties of chlorophylls, DAD is the main detector used, although Raman spectroscopy was also proposed [26]. However, the similarity of the UV-Vis spectrum between several chlorophyll derivatives, made essential the utilization of mass spectrometry to properly identify the different substituents of the chlorophyll compounds [27,28]. Details of the mass spectrometry characteristics of the chlorophyll compounds is not very abundant, although several studies have been developed [23,27]. 

**Figure 4:**
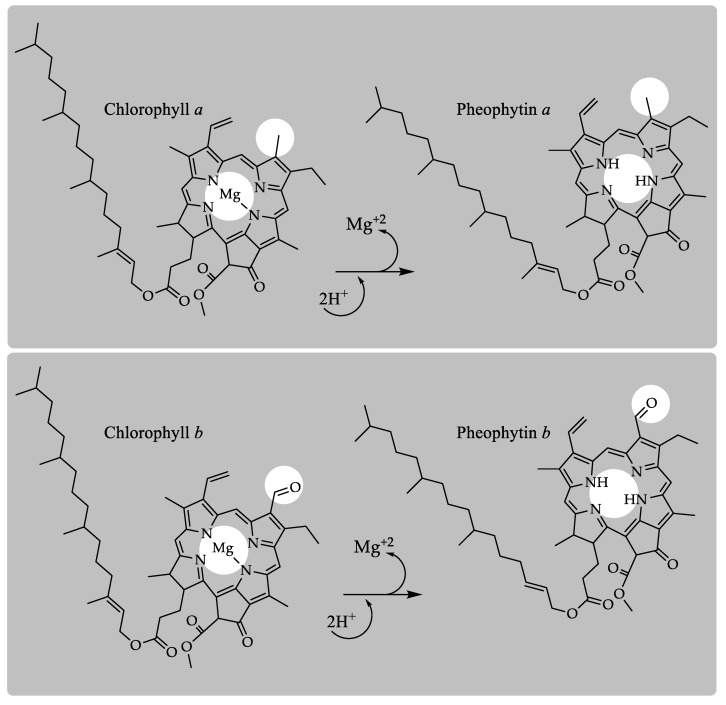
Chlorophyll derivatives present in the colorant E140i or “chlorophyll”.

The colorant named “chlorophyll” or “E140i”, as it is marketed after direct solvent extraction from edible green plant materials, is mainly composed of chlorophyll *a* and *b*, along with their corresponding pheophytin derivatives, which are originated during the extraction at low pH, which catalyzes the substitution of the central magnesium by hydrogens (Figure 4). The relative proportion of chlorophyll to pheophytin present in the colorant depends on the conditions of the manufacturing practices, and it is highly variable between the different suppliers. In addition, the chlorophylls present in the colorant product are very labile, and the chemical conditions during the food processing accelerate the degradative process. The transformation causes a change in color from green to brown; a collateral effect which is not desired by the food industry.

The natural green colorant E140ii is commonly named “chlorophyllin”. This term is the source of confusion, because currently there is no clear chemical definition of that term, yielding a gap between the genuine chemistry of chlorophylls and the industrial (commercial) application of the nomenclature. Originally, the term [29] referred to the chlorophyll derivatives produced after the saponification of chlorophyll without a change in color. At that stage, when the definitive structure of chlorophyll was not described yet, chlorophyllin exclusively comprised those chlorophyll derivatives arising after the phytol ester breakdown. This means that chlorophyllins keep the central magnesium ion intact. Later, this term increased its application to other chlorophyll derivatives produced during the saponification; i.e., those arising from the cleavage of the isocyclic ring. However, the exact chemical definition of chlorophyllins covers those chlorophyll derivatives with an intact central magnesium ion. Therefore, the present commercial (industrial and sometimes legislative) use of the term “chlorophyllins” is incorrect, because the colorant products include chlorophyll structures without magnesium [30]. 

**Figure 5:**
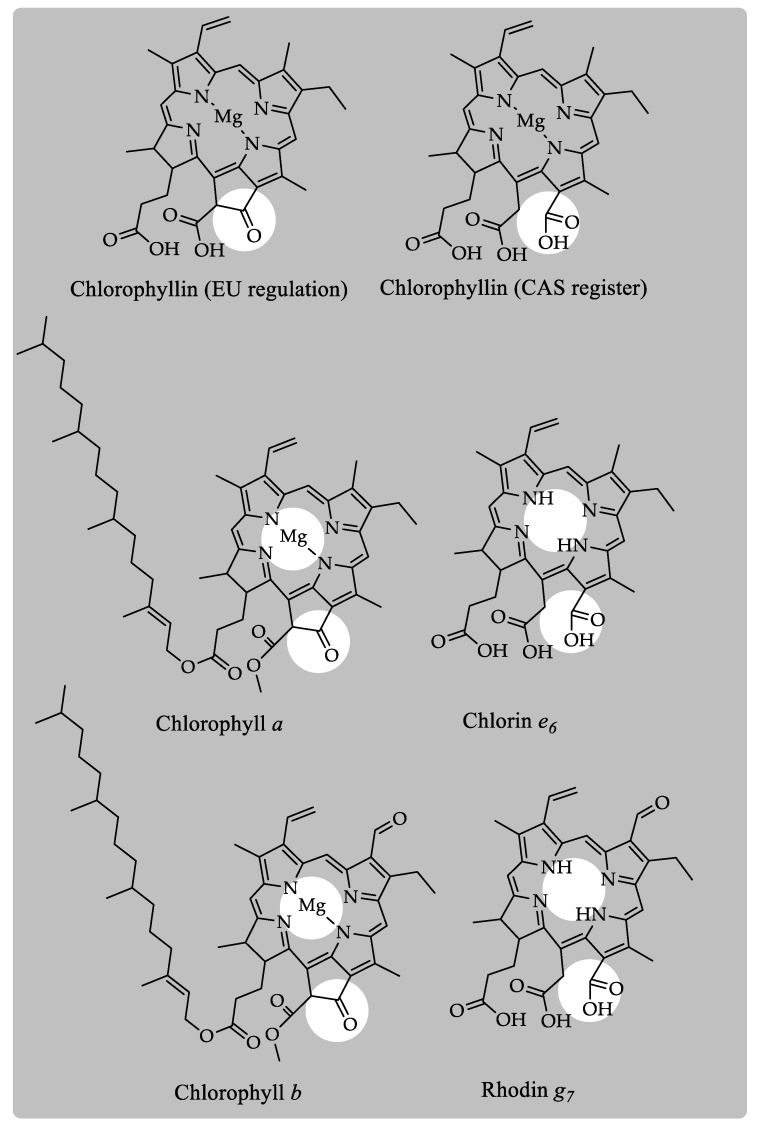
Chlorophyll derivatives present in the colorant E140ii or “chlorophyllins”.

Figure 5 describes some of the different chlorophyll structures that have been assigned to the “chlorophyllin *a*” denomination. For example, EU regulation [8] describes a structure with elemental composition C_34_H_32_MgN_4_O_5_ and molecular weight 600.9467 Da that chemically corresponds to demethylated chlorophyllide *a*. This compound originates from chlorophyll after the breakdown of the phytyl ester bond at C17^3^ (chlorophyllide *a*) and the fragmentation of the methoxy group at C13^1^. Other examples are the compound described in the Chemical Abstract Service (CAS 15611-43-5) or in PubChem (123798), which chemically corresponds with magnesium chlorin *e*_6_. This structure with an elemental composition C_34_H_34_MgN_4_O_6_ and molecular weight 618.962 Da is formed from chlorophyll *a* after the fragmentation of the phytyl ester bond, loss of the carboxymethoxy group and opening of the ring V. However, during chlorophyll saponification, the reaction required for E140ii manufacturing, the central magnesium ion is replaced by two hydrogens. Consequently, the main chlorophyll compounds that form part of the colorant E140ii are the chlorin *e*_6_ which originates from chlorophyll *a* and the rhodin *g*_7_ formed from chlorophyll *b*, besides other chlorophyll derivatives such as pheophorbides.

**Figure 6:**
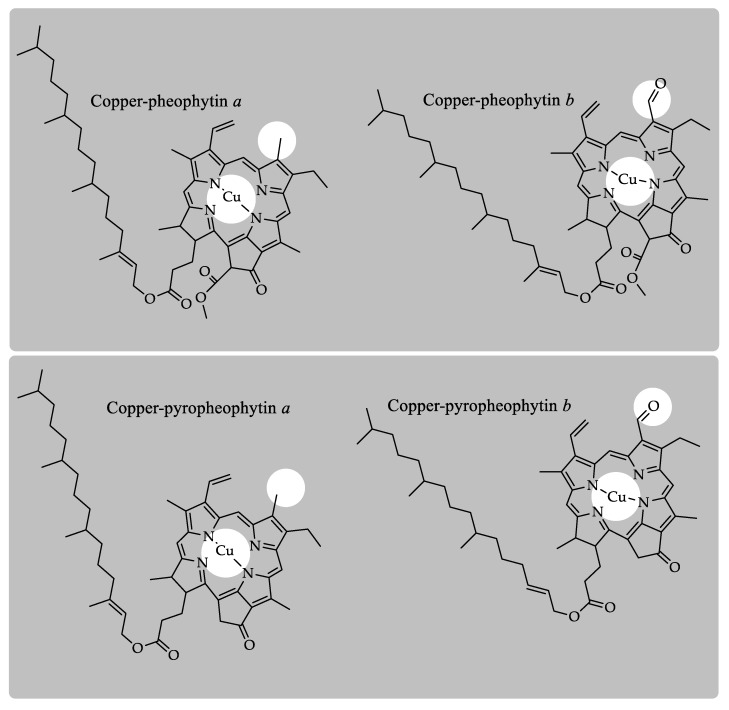
Chlorophyll derivatives present in the colorant E141i or “copper-chlorophylls”.

The colorant E141i (Figure 6) is known as “copper chlorophyll”, although it would be more accurate to label it as pheophytin with copper. The former nomenclature aims to denote the oily consistency of the colorant, which in chemical terms means that it is constituted by phytylated chlorophyll derivatives and, therefore, is lipid-soluble. This premise, in conjunction with the required copper treatment for its production, means that the main chlorophyll derivatives present in the colorant E141i are copper–pheophytin derivatives. In fact, an HPLC method was developed for the detection and quantification of copper chlorophylls *a* and *b* (copper pheophytins *a* and *b*) [25]. Later, several methods have been settled with the same aim [24,31]. At any rate, the complete characterization of several commercial E141i samples showed not only differences in the chlorophyll profile between them, but also that the common main compound present in all samples is copper–pyropheophytin *a* [32]. The MS characterization of this chlorophyll derivative has allowed us to propose a specific product ion as a probe for tracking the presence of the E141i food where its use is not authorized, particularly in olive oil [28]. Other chlorophyll derivatives that could be present in this colorant are copper–pheophytin *a*, copper–pheophytin *b*, copper–pyropheophytin *b*, and some others in trace amounts [32].

**Figure 7:**
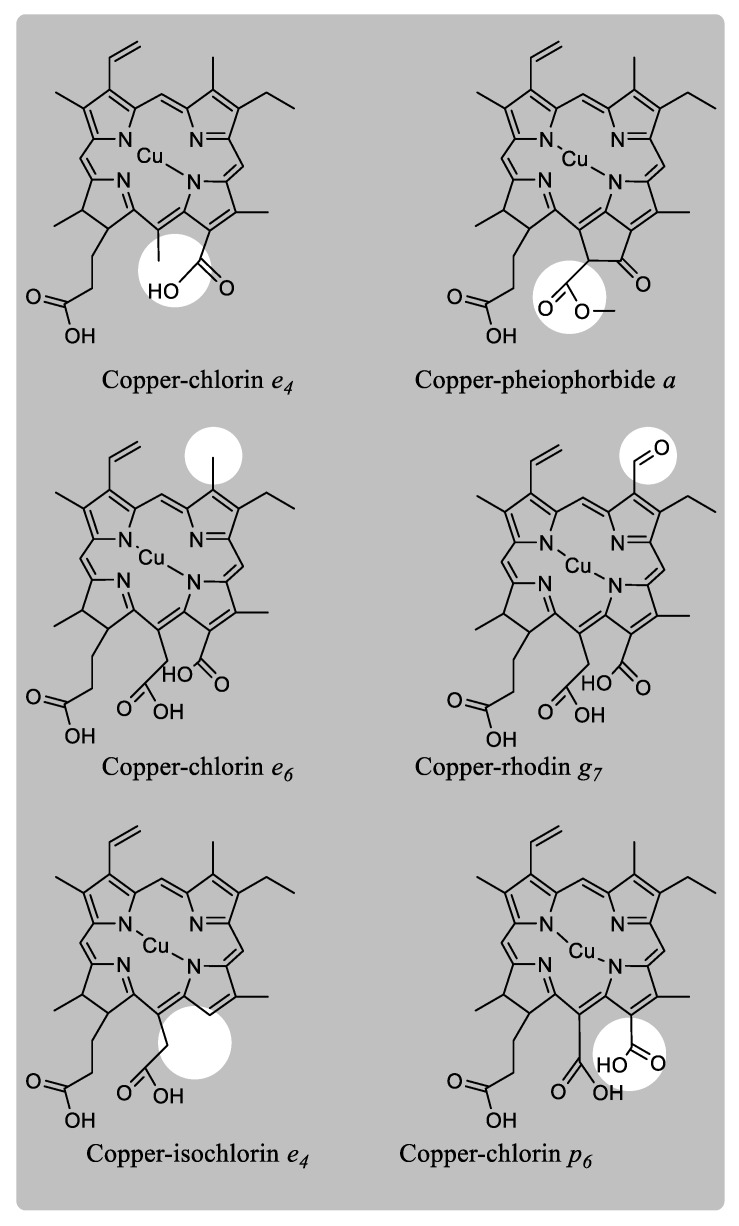
Chlorophyll derivatives present in the colorant E141ii or “copper-chlorophyllins”.

The water-soluble green food colorant E141ii (Figure 7) has been the most studied as it is one of the most popular within the food industry. Its manufacture requires, besides solvent extraction, saponification and treatment with copper salts, which means a higher degree of transformation from chlorophylls. The first chlorophyll derivative identified in this colorant was the copper chlorin *e*_4_ [33]. 

Following this, three additional new chlorophyll derivatives (copper pheophorbide *a*, copper chlorin *e*_6_ and copper rhodin *g*_7_) were identified as the main components in addition to copper chlorin *e*_4_, in the commercial “sodium copper chlorophyllin” (SCC), by comparison with authentic standards [34]. Authors determined not only absorption maxima but also molar extinction coefficients. Later, in five industrial samples of copper chlorophyllins, copper isochlorin *e*_4_ was identified as the major chlorophyll derivative in most of the colorants [35], describing a high variability between commercial preparations. Different manuscripts have dealt with the presence of this colorant in processed foods [18,24,36,37,38,39]. At any rate, a further advance was the single HPLC-MS analysis published to date [27] of the chlorophyll derivatives present in several commercial colorants. The authors identified several new chlorophyll structures, such as Cu-chlorin *p*_6_, although additional assignments were tentatively made. The lack of authentic standards and the fact that most of the substituents in the chlorophyll tetrapyrrole do not modify the features of the UV-Vis spectrum (chlorin/isochlorin, methyl/ethyl, ester or not, etc.) makes the correct identification of chlorophyll derivatives exclusively by their chromatographic properties difficult, or even leads to the misidentification of the chlorophyll compounds being possible.

## 4. In Vivo and In Vitro Adsorption, Distribution, Metabolism and Excretion (ADME)

In spite of the widespread presence of natural green colorants in foodstuffs, there is a general lack of information regarding their ADME. Although few studies have been developed either in vitro or in vivo with the aim of stablishing the ADME of E140i (chlorophylls) and E141ii (“copper chlorophyllin”), there are no data regarding the bioavailability of E140ii (chlorophyllins) and E141i (copper chlorophylls). Consequently, there are still several open questions about the in vivo ADME of natural green colorants. 

### 4.1. ADME of E140i 

At the in vitro level, the first study dealing with the digestion of green vegetables and subsequent absorption of the micellarized pigments by human intestinal Caco-2 cells was reported in 2001 [40]. The tested foods included fresh spinach puree, heat and acid-treated spinach puree, and spinach puree treated with ZnCl_2_. The original chlorophylls were transformed into pheophytins during digestion as a consequence of the gastric pH. However, pheophytins with zinc were relatively stable during the simulated digestion. The authors demonstrated that around 5–10% of the micellarized pheophytins were absorbed by the cells and that micellarization and uptake changed significantly between the different kinds of chlorophyll derivatives. This was the first hint at determining that micellarization and absorption are greatly influenced by the lipophilicity of the compounds. Specifically, the more lipophilic molecules showed the highest accumulation in the intestinal cells. 

Subsequently, these results were confirmed by applying the same in vitro protocol to pea preparations [41], pure standards of chlorophyll derivatives isolated from natural sources [42], and seaweeds [43]. Specifically, it has been shown in vitro that pheophorbides (dephytylated chlorophylls) are preferentially absorbed over pheophytins (phytylated chlorophylls) [42,43]. At this point, and by means of comparative absorption experiments with the Caco-2 cellular model at different concentrations of pheophytins, it was hypothesized that the absorption process could correlate with a passive facilitated mechanism [42]. On the contrary, to decipher the kind of cellular transport process for pheophorbides, Viera et al. [44] described the production of micelles rich in pheophorbide *a* to reach physiological micellar concentrations (7 μm). Pre-incubations of cell monolayers with different amounts of one specific inhibitor of the Scavenger Receptor class B type I (SR-BI) transporter (BLT1), significantly inhibited the uptake of pheophorbide *a*, which strongly suggests that SR-BI is involved in the transport of pheophorbide *a*. Consequently, the protein-mediated absorption of pheophorbide explains the preferential absorption of this chlorophyll derivative.

At the in vivo level, the pioneering studies on chlorophyll assimilation were based on the quantification of chlorophyll derivatives (E140i) in the feces of animals and by applying a balance matter (ingested against excreted) approach. Therefore, it has been historically assumed that chlorophylls were not absorbed by the organism, since almost all ingested chlorophylls were excreted. In this line, Brugsch and Sheard [45] estimated the quantitative decomposition of chlorophylls in the human body. Researchers supplied encapsulated crystalline chlorophyll to humans (100 mg per day for 4 days), showing that the highest percentage of degraded chlorophylls matched with fecal pheophytin [46]. Additional experiments [47] found scarce evidence of the subsequent hydrolysis of pheophytin to the water-soluble pheophorbide. The release of phytol by the colonic microflora in humans fed with pheophytin or spinach labelled with ^14^C led to the conclusion that the main final digestive products of the ingested chlorophylls were the pheophytins. The significance of this finding lies in the fact that chlorophylls are the single source of phytanic acid, which accumulates in the Refsum disease [47].

After a great period of time, the in vivo absorption of chlorophyll derivatives was investigated in dogs fed with a diet containing 73 mg of chlorophyll (spinach)/kg of diet for 10 days [48], obtaining an apparent absorption of 3.4% of chlorophylls (determination of ingested against excreted). A second experiment was also carried out increasing the dose of chlorophyll; that is, the dogs consumed a diet containing 10% of dried spinach for 10 days. Under these conditions, no chlorophyll derivatives could be detected in the peripheral blood until 150 min after consumption, which suggests to the authors that chlorophylls were hardly absorbed and/or readily metabolized and excreted through the bile. In addition, chlorophylls *a* and *b* were transformed into their corresponding pheophytin derivatives in the gastrointestinal tract, and the authors concluded that beyond pheophytins, no other degradation products were produced.

However, opposite to the classical hypothesis, the in vivo ADME experiments of E140i developed with 30 mice fed with a diet supplemented with spirulina for four weeks [42] showed for the first time the existence of a first-pass chlorophyll metabolism in animals. The analyzed livers accumulated a diverse profile of chlorophyll derivatives, which were identified by HPLC-MS^2^. The study highlighted that chlorophyll derivatives that retain the phytyl chain in their structure (apolar derivatives) are available for absorption from a dietary source and accumulate in the liver. Nevertheless, the explicit enrichment of the liver with pheophorbide *a* is particularly significant. Two possible mechanisms are proposed: that phytylated chlorophylls can be further metabolized in the liver to pheophorbide, or the existence of an intestinal transporter for this metabolite. If the pheophytin is de-esterified in the liver to yield pheophorbide, the authors reveal the enigmatic origin of phytol in the liver [49]. Indeed, as was mentioned above, the authors also presented data on the implication of the intestinal brush border transporter SR-BI in the absorption of pheophorbide. Therefore, independent of the exact mechanism, the present chlorophylls in the colorant E140i are at least absorbed, metabolized, accumulated and excreted in mammals. 

### 4.2. ADME of E140ii and E141i

There are no scientific data regarding the in vitro or in vivo absorption, distribution, metabolism, excretion and toxicity of chlorophyllins (E140ii) or copper chlorophylls (E141i). This lack of relevant data was also revealed through different Scientific Opinions [50,51,52] by the European Food Safety Agency (EFSA). According to the conclusions of the panel of experts of the EFSA and in the sight of the great interest of business operators, the European Commission launched on October 2017 a call for scientific and technical data requested by EFSA to complete the risk assessment. The collected data within a three–four-year timeframe will be assessed by the EFSA, and later the European Commission will take the final decision on the status of the revised colorants [53].

### 4.3. ADME of E141ii

Following an experimental design similar to the analysis of the ADME of E140i, the Schwartz group [54] showed that part of the chlorophylls present in the E141ii colorant was absorbed through the human intestine. In detail, four chlorophyll derivatives—copper rhodin *g*_7_, copper chlorin *e*_6_, copper chlorin *e*_4_ and copper pheophorbide *a*—from a commercial-grade sodium copper chlorophyllin were assayed. The copper chlorin *e*_4_ (the main component of E141ii) was highly stable to the simulated in vitro digestion process while most of the copper chlorin *e*_6_ was lost. However, the integration of the colorant in applesauce significantly improved the recovery of chlorin *e*_6_, demonstrating the protective role of the food matrix. Moreover, copper chlorophyll derivatives were efficiently absorbed by the intestinal epithelium cells, probably through an active transport. Part of these chlorophyll compounds were even detectable in the basolateral compartment, which means that they are ready to be transported to peripheral tissues.

The first in vivo studies of E141ii were carried out by Henderson and Long in 1941 [55], who orally administered natural chlorophyll and SCC to rats, discovering the existence of uncharacterized derivatives dispersed throughout the liver, lymph nodes and the spleen. Reber and Willigan (in 1954) [56] obtained with their studies the first in vivo indications about the absorption of E141ii. Rats fed with 1% of copper chlorophyllins in their diet for 15 weeks showed, after euthanasia, a greenish tone throughout the skeletal muscle of the body, indicating a systemic distribution of the chlorophyll derivatives. In the same year [57], a study with different doses of copper chlorophyllin in the diet of rats resulted in the transport through the gastrointestinal membrane and accumulation in plasma of copper chlorophyllins in the μg range. The authors did not detect any copper derivative in the organs, and they assumed that copper chlorophyllins are excreted in the feces. Later, the chemopreventive activity against tumorigenesis of copper chlorophyllin was tested in female mice [58]. In this study, the sodium salt of copper chlorophyllin administered orally was rapidly distributed to the heart, liver, skin, kidneys, and lungs. A subsequent study estimated the accumulation of dietary E141ii (10 or 30 mg/kg) in different organs of 30 Wistar rats [59]. The results showed that while copper chlorin *e*_4_ was effectively absorbed and accumulated in serum, liver and kidneys, copper chlorin *e*_6_ was not detected by HPLC analysis in sera or tissues, according to the data published by Ferruzzi et al. [54].

Indirect evidence in humans suggests that any type of absorption could take place following a copper chlorophyllin diet. For example, urine discoloration has been described with incontinent patients subjected to an oral copper chlorophyllin intake (100–200 mg/day) [60,61]. However, the single evidence of the in vivo absorption and accumulation of E141ii in humans was provided by the studies of Egner et al. [62]. Thus, 182 volunteers ingested 100 mg of copper chlorophyllins for 16 weeks and three times per day (copper chlorin *e*_4_, copper chlorin *e*_6_ and copper chlorin *e*_4_ ethyl ester). The study described for the first time the presence of copper chlorin *e*_4_ ethyl ester as well as copper chlorin *e*_4_, but not copper chlorin *e*_6_, in the sera of all the participants. Therefore, certain components are able to be absorbed through the gastrointestinal membrane. Probably, the instability of copper chlorin *e*_6_ to digestion could be responsible for the lack of appearance of this compound in human serum [63].

## 5. “Coloring Foodstuff”

The global trend of the replacement of synthetic colors with natural ones has created a new category in the market known as “coloring foodstuffs”. This class includes food ingredients such as fruits or vegetables whose secondary effect is coloring. This new conception is bringing regulatory problems as these substances are not covered by the current regulation on food additives in the EU. As the situation is rather unclear and there is an ongoing debate on the distinction between color additives and coloring foodstuffs [16], the European Commission (Standing Committee on the Food Chain and Animal Health) endorsed the Guidance notes providing a tool for classification for when a substance should be considered a color additive or not [64]. The distinction in this guidance is based on the extraction methodology: if the method is a non-selective extraction procedure, then the obtained product is a food ingredient, not a food additive. On the contrary, if the extraction is selective for obtaining a pigment, the compound is considered to be a color additive and consequently covered by the regulation on food additives. Previous to this guidance, nettles and spinach were the preferred coloring foodstuff to provide green hues to foods. However, now these color solutions no longer satisfy the criteria of EU guidelines. Otherwise, blends with spirulina are the alternative to create brilliant shades of green for confectionary products and ice creams; for example, with safflower. Specifically, in the USA, spirulina derived from *Arthrospira platensis* has been recently added to the list of approved color additives exempt from certification in response to a Mars Inc. petition [16]. In relation to spirulina extract, the Joint FAO/WHO Expert Committee has requested, in the meeting held in June 2018, information on the products on the market by December 2019 in order to remove the tentative designation from the specifications. Specifically, it is necessary to provide data on the full compositional characterization of commercial products and regarding the validated analytical methods applied for the identification of the substance and for the determination of the purity of the substance.

In any case, there is a new growing sector in the market, with several different extracts sold by different suppliers. Table 4 list the coloring foodstuffs authorized in the EU and USA, describing the maximum amounts and the foods allowed. As they are considered “natural”, to date, no amount limitations have been defined, and in the EU, as they are classified as ingredients, there are no restrictions for their use in any food category.

As a consequence of the natural trend in the coloring food market, recent research has developed looking for new strategies to develop new “natural” green colorants. For example, the utilization of an extract from the leaves of *Centella asiatica* L. after steaming and metal complexations has been proposed [15]. The authors assessed the stability and cytotoxicity in different beverages and food models as an alternative to synthetic colorants. Following the same aim, the properties of spray-dried microalgae have been analyzed (color, sensory and textural qualities) as a natural coloring agent in chewing gum [65]. Although positive results have been obtained with *Isochrysis galbana* and *Nannochloropsis oculata*, the spray-dryer technique requires an optimization in the base of the characteristics of each microalgae specie.

## 6. Stabilization of Green Color

Chlorophylls are stable pigments in their natural environment in physiological conditions. However, once extracted or processed with changes in pH value and temperature during the processing and storage of green foods, chlorophylls are prone to experience modifications in their structure, which consequently change their chromatic properties. Probably, the main reaction that affects chlorophylls is the substitution of the central magnesium ion by two hydrogens. The significance of the reaction for the food industry is related with the drastic change in color, because magnesium-derivatives are green, while magnesium-free derivatives (mainly pheophytins and pheorphorbides) are brown. Consequently, this easy and fast reaction is the principal cause of the loss of the original green color during the processing and storage of green foods. As has been stated before, the reduction or even withdrawal of the original green color is associated by the consumer with a decrease in the quality of the product. Therefore, the food industry has developed several strategies to preserve the initial green coloration. The early attempts consisted of the addition (even in-the-can coating) of alkalinizing agents, but the appearance of negative secondary effects such as a softening of the edible material or an off-flavor release [6] made them to fall into disuse. 

A second historical approach has been the substitution of the central hydrogen atoms by zinc or copper ions to form the more stable green metallochlorophylls which consequently “re-green” the corresponding food product. The conditions for the production of zinc and copper complexes of chlorophyll derivatives in vegetables during processing have been optimized through the years [66,67,68]. Due to the industrial and commercial importance of this process, numerous patents have been published, with the most known being the so-called “Veri-green” method [69]. This patent was developed by the former Continental Can Company and consisted of the blanching of vegetables in brine solutions with some amounts of Zn^+2^ or Cu^+2^ salts to form mainly zinc or copper pheophytins [70] and make the processed vegetables greener. However, the patent was unproductive because the maximum limit for zinc concentrations established by the FDA is 75 ppm, and higher amounts of zinc are required to yield an acceptable and desirable green color. To overcome this limitation, new strategies for the encapsulation of zinc-chlorophylls are currently under development using different matrices such as gum Arabic, maltodextrin and OSA modified starch [71] or whey proteins [72]. Indeed, different techniques are being set up to microencapsulate raw chlorophyll extractions from edible vegetables without any salt treatment. For example, spray drying is a dehydration process which has been successfully applied to encapsulate other food compounds, and recently, the physicochemical properties of the process have been optimized to form whey protein isolate–kale leaf chlorophyll microcapsules [73]. A further step in the use of this procedure as an available alternative for the food industry is the enhancement of the stability of green pigments for heated foods. For example, the stability of alfalfa leaves microencapsulated towards temperature regimes and pH conditions has been determined [74], obtaining optimal conditions.

## 7. Conclusions

The market of the food colorants is evolving to more natural formulations, which means the appearance of new definitions and consequently, the necessity of new legislations. Green food colorants are one of the most affected. First, because natural green colorants are very labile and secondly, because it is not easy to reproduce green hues naturally. Consequently, in the near future the consumer will face new compounds responsible of the green color in foods. At present, independently of the existence of serious problems due to different legislations and different definitions between countries, authorized green natural colorants are incompletely characterized. This review has summarized the present knowledge of the compounds comprised the authorized green colorants, highlighting the common errors in their daily management. Finally, the scarce research developed on the ADME of green authorized food colorants is also considered but with the confidence that new advances will be shortly achieved.

## Figures and Tables

**Table 1:** Classification of authorized natural green colorants according to different regulations.

Country	Chlorophyll	Chlorophyllin	Cu-Chlorophyll	Cu-Chlorophyllin	Na-Fe-Chlorophyllin
EU	E140i	E140ii	E141i	E141ii	
USA				73.125	
Japan	177	116	266	265	257
India	6				
China			08.153	08.009	
CA ^1^	INS 140	INS 140	INS 141i	INS 141ii	

^1^ CA: Codex Alimentarius.

**Table 2:** Classification of authorized artificial green colorants by different legislations.

Denominations	Green S	Fast Green FCF
CI ^1^	44090	42053
EU	E142	-
USA	-	FD&C green No. 3
Japan	-	Food green No. 3
India	-	Fast green FCF
China	-	-
CA	INS 142	INS 143

^1^ The Color Index^TM^ is a classification system for dyes and pigments globally used by manufacturers, researchers and users of dyes and pigments (https://colour-index.com/).

**Table 3:** Main chlorophyll derivatives in function of their substituents.

Chlorophyll Compound	R_1_	R_2_	R_3_	R_4_
Chlorophyll *a*	Mg	CH_3_	phytyl	COOCH_3_
Chlorophyll *b*	Mg	CHO	phytyl	COOCH_3_
Chlorophyllide *a*	Mg	CH_3_	H	COOCH_3_
Pheophytin *a*	H	CH_3_	phytyl	COOCH_3_
Pheophytin *b*	H	CHO	H	COOCH_3_
Pheophorbide *a*	H	CH_3_	H	COOCH_3_
Pyropheophytin *a*	H	CH_3_	phytyl	H

**Table 4:** Classification of “coloring foodstuff” in EU and USA.

	Vegetable Juice	Fruit Juice	Spirulina Extract
**European Union**			
	+	+	+
Allowed foods	Foods in general	Foods in general	Foods in general
Maximum limit	*Quantum satis*	*Quantum satis*	*Quantum satis*
**United States**			
Denomination	73,260	73,250	73,530
Allowed foods	Foods generally	Foods generally	Confections (including candy and chewing gum), frostings, ice cream and frozen desserts, dessert coatings and toppings, beverage mixes and powders, yogurts, custards, puddings, cottage cheese, gelatin, breadcrumbs, ready-to-eat cereals (excluding extruded cereals), coating formulations applied to dietary supplement tablets and capsules
Maximum limit	GMP ^1^	GMP	GMP

^1^ GMP: good manufacturing practices.
